# Supplementary material for: Empirical methods for controlling false positives and estimating confidence in ChIP-Seq peaks
Source: BMC Bioinformatics. 2008 Dec 5;9:523. doi: 10.1186/1471-2105-9-523 (PMC2628906; doi:10.1186/1471-2105-9-523)
Supplement: Additional File 1 — A variety of html documents from the USeq web site detailing the available applications, their best usage, output file type descriptions, command line menus, etc. [file 1471-2105-9-523-S1.zip › USeqUserGuides/JavaDoc.zip!/JavaDoc/util/bio/seq/FetchIndexedSequence.html]

FetchIndexedSequence


|  |  |  |
| --- | --- | --- |
| **Overview** \| **Package** \| **Class** \| **Use** \| **Tree** \| **Deprecated** \| **Index** \| **Help** | |  |
| **PREV CLASS**   **NEXT CLASS** | **FRAMES**    **NO FRAMES**     **All Classes** |  |
| SUMMARY: NESTED \| FIELD \| CONSTR \| METHOD | DETAIL: FIELD \| CONSTR \| METHOD |  |


---


## util.bio.seq Class FetchIndexedSequence

```
java.lang.Object
  util.bio.seq.FetchIndexedSequence
```

---

``` public class FetchIndexedSequence extends java.lang.Object ```

Fetches sequence given a directory of indexed sequences, see IndexFastas.
Start coordinates are always zero based; end coordinate is excluded if interbase is true,
or included if false.

---

**Constructor Summary**

`FetchIndexedSequence(java.io.File indexDirectory,
boolean interbaseCoordinates)`


| **Method Summary** | |
| --- | --- |
| `java.lang.String` | `fetchSequence(java.lang.String chromosome, int start, int stop)`             Returns null if chromsome doesn't exist or the start or stop indexedSequence out of range. |
| `java.lang.String[]` | `fetchSequences(int[][] startStop)`             Assumes startStop[index][0 start, 1 stop] is sorted by starts. |
| `java.lang.String[]` | `fetchSequences(java.lang.String chromosome, int[][] startStop)`             Returns null if chromsome doesn't exist or the start or stop indexedSequence out of range. |
| `static void` | `main(java.lang.String[] args)` |
| `void` | `makeIndexedSequences()` |
| `void` | `nullIndexedSequences()`             Good to call after each call to fetchSequences() unless you'll be fetching more from the same chromosome. |
| `boolean` | `setIndexedSequence(java.lang.String chromosome)`             Loads indexes for a particular chromosome. |

**Methods inherited from class java.lang.Object**

`equals, getClass, hashCode, notify, notifyAll, toString, wait, wait, wait`

**Constructor Detail**

### FetchIndexedSequence

```
public FetchIndexedSequence(java.io.File indexDirectory,
                            boolean interbaseCoordinates)
```


**Method Detail**

### setIndexedSequence

```
public boolean setIndexedSequence(java.lang.String chromosome)
```

:   Loads indexes for a particular chromosome. Nulls old sequences. Call before fecthSequences()

---


### fetchSequences

```
public java.lang.String[] fetchSequences(int[][] startStop)
```

:   Assumes startStop[index][0 start, 1 stop] is sorted by starts.
    Assumes indexedSequence is set!
    Must call setIndexedSequence(chromosome) prior to calling.

---


### fetchSequence

```
public java.lang.String fetchSequence(java.lang.String chromosome,
                                      int start,
                                      int stop)
```

:   Returns null if chromsome doesn't exist or the start or stop indexedSequence out of range.
    For multiple fetches use the fetchSequences() method.

---


### fetchSequences

```
public java.lang.String[] fetchSequences(java.lang.String chromosome,
                                         int[][] startStop)
```

:   Returns null if chromsome doesn't exist or the start or stop indexedSequence out of range.
    Assumes the startStop int[index][0 start, 1 stop] is sorted by starts.

---


### makeIndexedSequences

```
public void makeIndexedSequences()
```

---


### nullIndexedSequences

```
public void nullIndexedSequences()
```

:   Good to call after each call to fetchSequences() unless you'll be fetching more from
    the same chromosome.

---


### main

```
public static void main(java.lang.String[] args)
```


---


|  |  |  |
| --- | --- | --- |
| **Overview** \| **Package** \| **Class** \| **Use** \| **Tree** \| **Deprecated** \| **Index** \| **Help** | |  |
| **PREV CLASS**   **NEXT CLASS** | **FRAMES**    **NO FRAMES**     **All Classes** |  |
| SUMMARY: NESTED \| FIELD \| CONSTR \| METHOD | DETAIL: FIELD \| CONSTR \| METHOD |  |


---
